# Physical activity outside of structured therapy during inpatient spinal cord injury rehabilitation

**DOI:** 10.1186/s12984-016-0208-8

**Published:** 2016-11-15

**Authors:** Dominik Zbogar, Janice J. Eng, William C. Miller, Andrei V. Krassioukov, Mary C. Verrier

**Affiliations:** 1 Rehabilitation Research Program, Vancouver Coastal Health Research Institute, 4255 Laurel Street, V5Z 2G9 Vancouver, BC Canada; 2 International Collaboration on Repair Discoveries, Vancouver, Canada; 3 Department of Physical Therapy, Faculty of Medicine, University of British Columbia, Vancouver, Canada; 4 Department of Occupational Science & Occupational Therapy, Faculty of Medicine, University of British Columbia, Vancouver, Canada; 5 Division of Physical Medicine and Rehabilitation, Faculty of Medicine, University of British Columbia, Vancouver, Canada; 6 University Health Network-Toronto Rehabilitation Institute, Toronto, Canada; 7 Department of Physical Therapy, Faculty of Medicine, University of Toronto, Toronto, Canada

**Keywords:** PARA-SCI, Accelerometry, Physical activity, Spinal cord injury, Inpatient, Rehabilitation

## Abstract

**Background:**

Little information exists on the content of inpatient rehabilitation stay when individuals with spinal cord injury (SCI) are not engaged in structured rehabilitation therapy sessions. Investigation of inpatient therapy content is incomplete without the context of activities outside of this time. We sought to quantify physical activity occurring outside of physical therapy (PT) and occupational therapy (OT) sessions during inpatient SCI rehabilitation and examine how this activity changes over time from admission to discharge.

**Methods:**

In this longitudinal observational study at two inpatient SCI rehabilitation centres, 95 participants were recruited through consecutive admissions. Physical activity at admission and discharge was recorded by 1) self-report (PARA-SCI questionnaire) and 2) real-time accelerometers worn on the dominant wrist, and hip if ambulatory. For analyses, we separated participants into those with paraplegia or tetraplegia, and a subgroup of those ambulatory at discharge. Wilcoxon signed rank tests (admission vs. discharge) were used for PARA-SCI minutes and accelerometry activity kilocounts.

**Results:**

There was no change in self-report physical activity, where the majority of time was spent in leisure time sedentary activity (~4 h) and leisure time physical activity at a higher intensity had a median value of 0 min. In contrast, significant increases in physical activity outside PT and OT sessions from admission to discharge were found for wrist accelerometers for individuals with tetraplegia (i.e., upper limb activity) and hip accelerometers for ambulatory individuals (i.e., walking activity).

**Conclusion:**

Physical activity is low in the inpatient SCI rehabilitation setting outside of structured therapy with a substantial amount of time spent in leisure time sedentary activity. Individuals appear to have the capacity to increase their levels of physical activity over the inpatient stay.

## Background

Physical activity after a spinal cord injury (SCI) is important for optimizing recovery from SCI as well as the ability to improve secondary complications like physical deconditioning resulting from bed rest, cardiovascular disease and autonomic disorders [1]. Rehabilitation is effective in accelerating and promoting improvement in activities of daily living; indeed, a delay in starting appropriate and intensive activities may negatively influence a participant’s ultimate functional capability since the degree of post-SCI deconditioning will increase with a longer delay in starting an exercise program [2, 3].

There is some debate as to whether the level of physical activity during rehabilitation stay is adequate for optimizing neurological recovery or for achieving sufficient physical capacity for returning to the community [4, 5]. How much inpatient rehabilitation prepares individuals with SCI to engage in physical activity once they return home is unknown, though the significant decrease in physical activity that follows discharge [6] suggests preparation is not optimal. We have recently shown that the amount of cardiovascular stress experienced during physical therapy (PT) and occupational therapy (OT) is negligible [7], despite these being the most active times of the day according to participant self-report [8]. As time in therapy makes up only a small proportion of a patient’s day, it is important to develop an understanding of physical activity levels outside of rehabilitation therapy sessions in order to put time spent in PT and OT in context as well as assess the overall daily physical activity that the patient is experiencing. While some studies have evaluated therapy intensity or content during structured therapy [6, 9–14] this study is unique in that it captures physical activity outside of structured rehabilitation sessions and does so using both a self-report interview and a real-time, objective measure (accelerometry).

This study had two objectives. Objective 1: To quantify physical activity during inpatient rehabilitation outside of PT and OT sessions. Objective 2: To examine how or if physical activity outside of structured therapy changes over time from admission to discharge. We hypothesized that physical activity would be low, but would increase from admission to discharge.

## Methods

### Participants

Participants were a consecutive sample of traumatic and non-traumatic SCI admissions to inpatient care at two Canadian rehabilitation centres in two provinces. Nontraumatic SCI was defined as SCI resulting from spinal stenosis, tumour, ischemia, transverse myelitis, and infection [15]. Ambulatory participants were defined as those who were independently ambulatory (with or without assistive devices) at the time of the discharge assessment. Participants were excluded if they had a traumatic brain injury that significantly affected the content and delivery of therapy, if consent could not be obtained within the first week of admission, or if their length of stay in rehabilitation was projected to be less than 4 weeks as it precluded the ability to collect admission and discharge data.

**Fig. 1:**
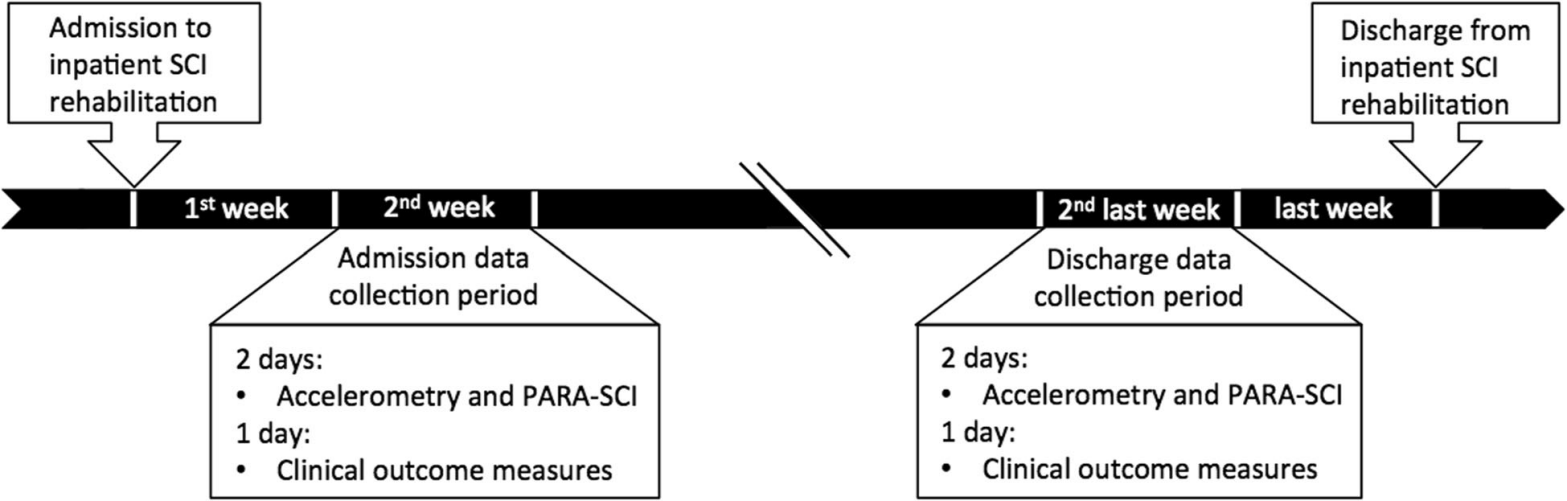
Data collection protocol

As displayed in Fig. 1, data were collected over 2 weekdays in the second week after admission and over 2 weekdays in the second-last week before discharge to minimize bias from admission and discharge assessments and discharge planning activities. On each data collection day, a research assistant met the participants in their rooms in the morning prior to breakfast before they had transferred from bed. At this time the participant put on the accelerometers and was reminded that they would be required to recall the events of their day that evening. In the evening of each day, when participants had transferred back to bed, the research assistant returned to collect the accelerometers and to administer the self-report physical activity questionnaire. In addition, information on the time of day when PT and OT sessions occurred was collected.

Approval for this study was obtained from the local university and health ethics boards and all participants provided informed consent before study enrolment.

### Physical activity measures

Actical accelerometers (Actical; Mini Mitter Co., Bend, OR) worn on the dominant wrist like a wrist watch quantified the amount and intensity of upper extremity activity using mean total activity kilocounts per day. The Actical accelerometer consists of a piezoelectric sensor that is responsive to acceleration, and hence deformation of the sensor results in a proportional charge. The signal is full wave rectified (i.e., absolute acceleration value) and filtered at a frequency range of 0.3–3 Hz, and then analog-to-digitally converted at a sampling rate of 32 Hz. The unit is sensitive to 0.05–2.0 G-force. The accelerometer record is integrated over 15 s as activity counts. Acceleration is detected in all three planes, although there is greater weighting for vertical accelerations. The specific algorithm to convert to activity counts is proprietary to Actical, although conversion from Actical counts to other accelerometer brands [16] as well as to estimates of energy expenditure has been established [17]. Increased activity counts may indicate longer wear time, more movement, and/or greater intensity of movement. Ambulatory individuals also wore an accelerometer on the right hip secured with a waist strap to detect the number of steps using the step-count function of the accelerometer. Wrist and hip accelerometers have shown test retest reliabilty coefficients of 0.89 and 0.74, respectively over separate inpatient rehabilitation days [18].

Participants completed the Physical Activity Recall Assessment for People with SCI (PARA-SCI), found valid and reliable in community dwelling individuals with SCI [19, 20]. This self-report questionnaire is administered via a semi-structured interview. The PARA-SCI measures the amount of physical activity individuals with SCI accumulate over a day and provides an estimate of time (in minutes) spent participating in mild, moderate and heavy intensity physical activities, as well as activities with no intensity (“nothing at all”) [21].

For the purpose of this study, the four intensities of the PARA-SCI were binned into two categories: ‘lower intensity’ comprising nothing or mild intensity and ‘higher intensity’ comprising moderate or heavy intensity. Moderate and heavy physical activity are intensities recommended by exercise guidelines for accruing health benefits in able-bodied healthy adults [22] and individuals with SCI [23]. We also reported seven categories in the PARA-SCI for the minutes of physical activity undertaken for: 1) the total time the accelerometer was worn, 2) any activities outside of PT and OT sessions, and then subcategories of 3) leisure-time sedentary activity (e.g., watching TV, playing board games, talking to friends/family, etc.), 4) activities of daily living (ADLs), (i.e., tasks which included feeding, transfers, toileting, bathing, dressing, walking or propelling a wheelchair), 5) appointments and educational/sedentary classes, 6) active group classes (organized classes including wheelchair skills, pulley, swimming pool, and hand classes), and 7) leisure-time physical activity (any physical activity intentionally engaged in by the participant outside of formal therapy times that is not an ADL).

### Clinical outcome measures

Clinical outcome measures of upper and lower extremity function were collected on a separate day within the admission and discharge data collection periods.

Grip strength was tested using a hand held Jamar Dynamometer (Nicholas MMT, Lafayette Instrument, Lafayette, IN). Participants performed 3 maximal voluntary contractions, with at least 30 s of rest between trials. The 3 trials were averaged to obtain a mean score in kilograms. All measurements were taken with the participant seated, with the elbow bent at 90° and the hand in a neutral position. This test has proven reliable and valid for assessing manual grip in both healthy and hand-injured populations [24, 25].

The Graded Redefined Assessment of Strength, Sensibility and Prehension (GRASSP) was used with participants with tetraplegia to evaluate the muscle, sensory, and grasping function of study participants. The GRASSP involves scoring 6 functional tasks, assessing upper extremity strength via muscle testing, and assessing sensibility of the hands using monofilaments. Test scores are summed for a total score for each hand (ranging from 0 to 116) with higher scores indicating better hand function [26]. The GRASSP has demonstrated reliability and validity in the SCI population [27].

Participants with ambulatory ability were assessed with the Walking Index for Spinal Cord Injury (WISCI II), which gauges locomotor performance on a 0 to 20 hierarchical scale where higher scores indicate better ambulatory ability and accounts for the requirement of devices, braces, and physical assistance used to complete a 10-m distance. The WISCI II is reliable and valid in the SCI population [28].

The 10 Meter Walk Test (10MWT) is a measure of functional capacity. For this test, ambulatory participants walk 14-m while being timed at their comfortable pace and at their maximal pace. The first and last 2 m are eliminated from the speed calculation to negate acceleration/deceleration effects [29]. The 10MWT has been shown to have excellent reliability and validity in incomplete SCI [30].

Also, descriptive information was collected for age, gender, plegia type (paraplegia/tetraplegia), aetiology (traumatic or nontraumatic), American Spinal Injury Association Impairment Scale grade, [31] length of stay in acute care and length of stay in rehabilitation.

### Data analyses

Descriptive statistics (means, standard deviations, medians, frequencies) for participant demographics, injury characteristics, and length of stay are included in Table 1. Wrist activity kilocounts, number of steps, and self-reported physical activity values were calculated by averaging measures collected over 2 days to obtain representative values of daily inpatient rehabilitation stay. Total day values (including time in and outside of PT and OT sessions) are included in Tables 3, 4 and 5 for context only. For analysis we separated data by plegia type (tetraplegia and paraplegia) and ambulation status (participants who were able to ambulate by the time of their discharge assessment).

**Table 1:** Demographic and SCI information for all patients and subgroups

Variable	All Patients	Paraplegia	Tetraplegia	Ambulatory^a^
n	95	53	42	33
Gender (M/F)	68/27 (72/28)	37/16 (70/30)	31/11 (74/26)	24/9 (73/27)
Traumatic/nontraumatic	66/29 (70/30)	32/21 (60/40)	34/8 (81/19)	23/10 (70/30)
Discharge AIS grade (A/B/C/D)^b^	23/12/12/48 (24/13/13/50)	13/6/9/25 (25/11/17/47)	10/6/3/23 (24/14/7/55)	1/2/0/30 (3/6/0/91)
Age (years)	49 ± 18, 53	48 ± 18, 52	51 ± 17, 54	51 ± 17, 53
LOS in rehabilitation (days)	97 ± 46, 95	86 ± 38, 76	112 ± 51, 118	75 ± 44, 63
LOS in acute care (days)	39 ± 39, 26	32 ± 33, 22	48 ± 45, 31	20 ± 13, 16

*n* number of patients, Values are *n* (%) or mean ± 1SD, median; *AIS* American Spinal Injury Society Impairment Scale, *LOS* length of stay

^a^The Ambulatory group is composed of a subset of all patients who were able to ambulate by the time of the discharge assessment

^b^While the AIS is valid for traumatic SCI, it has not been validated in non-traumatic SCI

To quantify changes from admission to discharge from inpatient SCI rehabilitation, Wilcoxon signed rank tests were used for wrist accelerometry, step counts, self-reported physical activity for lower intensity and higher intensity, and clinical outcome measures at admission and discharge. The *Z* value and effect size (*r* = *Z*/√n) (0.1 = small effect, 0.3 = moderate effect, 0.5 = large effect) [32] are documented. Reported values are medians unless stated otherwise.

Statistical software, SPSS 17 (SPSS Inc, Chicago, IL USA) was used for the analysis. A Benjamini-Hochberg correction (alpha value of 0.012) was calculated and employed to minimize the chance of type I error [33].

## Results

**Fig. 2:**
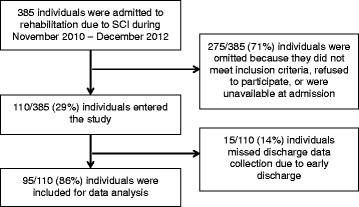
Flow diagram of recruitment to the study

Recruitment of participants is described in Fig. 2. Demographic information is presented in Table 1. There were statistically significant and clinically meaningful improvements for all clinical outcome measures (Table 2) from admission to discharge (*p* < 0.002) except grip strength for individuals with paraplegia.

**Table 2:** Clinical outcome measures at admission and discharge from inpatient rehabilitation

	Admission	Discharge	*Z value*	*p*	effect size
Particiapants with paraplegia					
Grip strength, (kg)	36, 23–47	39, 24–48	−0.87	0.38	−0.08
Participants with tetraplegia					
Grip strength, (kg)	0, 0–6.7	5.9, 0–17.2	−3.10	0.002*	−0.35
GRASSP	70, 33–98	87, 43–106	−3.76	<0.0001*	−0.48
Ambulatory Participants					
10MWT- comfortable, (m/s)	0.11, 0–0.65	0.77, 0.37–1.11	−4.60	<0.0001*	−0.59
10MWT- maximal, (m/s)	0.16, 0–0.87	1.04, 0.64–1.39	−4.51	<0.0001*	−0.58
WISCI II	8, 0–13	19, 13–20	−4.01	<0.0001*	−0.53

All values are median, Q1–Q3, *p ≤ 0.012 (Benjamini-Hochberg corrected significance level), *GRASSP* graded redefined assessment of strength, sensibility and prehension, 10*MWT* 10 meter walk test, *WISCI* II walking index for spinal cord injury II

### Self-reported physical activity

**Fig. 3:**
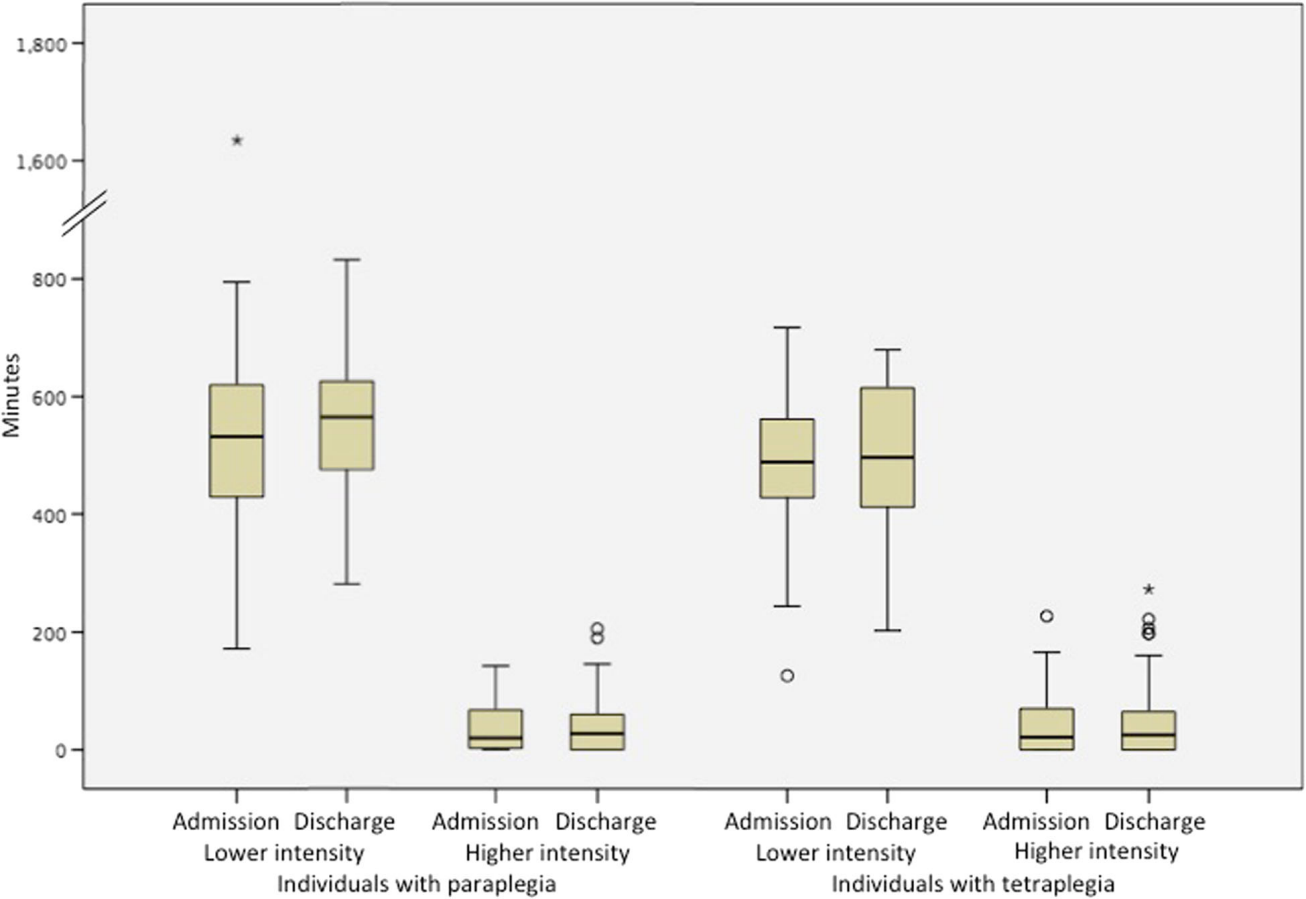
Self-reported minutes of physical activity at admission and discharge from inpatient rehabilitation

For the PARA-SCI, there was no statistically significant change over time in self-reported physical activity minutes outside therapy for both individuals with paraplegia and tetraplegia at lower and higher intensities (Fig. 3), and for any of the categories (e.g. ADL, leisure time physical activity, etc.) (Table 3, Table 4).

**Table 3:** Individuals with paraplegia: Self-reported minutes of physical activity at admission and discharge from inpatient rehabilitation

	Admission	Discharge	*Z value*	*p*	effect size
Minutes of all physical activity intensities					
Total Day (including PT/OT)	663, 567–755	671, 607–777	~	~	~
Outside PT/OT only	555, 468–657	587, 526–685	~	~	~
Leisure time sedentary activity	290, 183–371	275, 201–356	~	~	~
Activities of daily living	196, 153–243	209, 166–265	~	~	~
Appts, educational classes, etc.	36, 8–55	30, 5–68	~	~	~
Leisure time physical activity	10, 0–31	10, 0–51	~	~	~
Physically active group classes	0, 0–16	0, 0–30	~	~	~
Minutes of lower intensity physical activity					
Total Day (including PT/OT)	557, 459–649	580, 498–681	~	~	~
Outside PT/OT only	532, 410–626	565, 471–633	−1.84	0.066	−0.18
Leisure time sedentary activity	290, 183–371	275, 201–356	−0.62	0.54	−0.06
Activities of daily living	186, 141–213	193, 150–258	−1.50	0.13	−0.15
Appts, educational classes, etc.	36, 8–55	30, 5–68	−0.28	0.78	−0.03
Leisure time physical activity	0, 0–10	0, 0–11	−0.49	0.62	−0.05
Physically active group classes	0, 0–0	0, 0–18	−1.86	0.063	−0.18
Minutes of higher intensity physical activity					
Total Day (including PT/OT)	106, 72–133	93, 60–120	~	~	~
Outside PT/OT only	20, 2–68	28, 0–61	−0.42	0.68	−0.04
Leisure time sedentary activity	0	0	~	~	~
Activities of daily living	7, 0–31	0, 0–30	−0.86	0.39	−0.08
Appts, educational classes, etc.	0	0	~	~	~
Leisure time physical activity	0, 0–10	0, 0–26	−1.57	0.12	−0.15
Physically active group classes	0, 0–0	0, 0–0	−0.08	0.94	−0.01

All values are median,Q1-Q3; *p* ≤ 0.012 (Benjamini-Hochberg corrected significance level); *PT* physical therapy, *OT* occupational therapy, *Appts* appointments

**Table 4:** Individuals with tetraplegia: Self-reported minutes of physical activity at admission and discharge from inpatient rehabilitation

	Admission	Discharge	*Z value*	*p*	effect size
Minutes of all physical activity intensities					
Total Day (including PT/OT)	641, 558–690	679, 591–755	~	~	~
Outside PT/OT only	533, 462–592	556, 488–657	~	~	~
Leisure time sedentary activity	264, 182–324	235, 158–311	~	~	~
Activities of daily living	193, 157–241	215, 151–266	~	~	~
Appts, educational classes, etc.	30, 7–46	28, 0–62	~	~	~
Leisure time physical activity	0, 0–13	11, 0–30	~	~	~
Physically active group classes	0, 0–24	0, 0–31	~	~	~
Minutes of lower intensity physical activity					
Total Day (including PT/OT)	525, 442–582	558, 460–661	~	~	~
Outside PT/OT only	489, 426–563	497, 411–620	−1.01	0.31	−0.11
Leisure time sedentary activity	264, 182–324	235, 158–311	−0.46	0.65	−0.05
Activities of daily living	170, 139–211	197, 138–247	−1.24	0.21	−0.14
Appts, educational classes, etc.	30, 7–46	28, 0–62	−0.46	0.64	−0.05
Leisure time physical activity	0, 0–8	0, 0–6	−0.61	0.54	−0.07
Physically active group classes	0, 0–0	0, 0–23	−1.94	0.053	−0.21
Minutes of higher intensity physical activity					
Total Day (including PT/OT)	103, 66–138	96, 48–171	~	~	~
Outside PT/OT only	22, 0–71	26, 0–67	−0.92	0.36	−0.10
Leisure time sedentary activity	0	0	~	~	~
Activities of daily living	9, 0–32	10, 0–33	−0.11	0.91	−0.01
Appts, educational classes, etc.	0	0	~	~	~
Leisure time physical activity	0, 0–0	0, 0–21	−2.15	0.031	−0.24
Physically active group classes	0, 0–11	0, 0–0	−0.43	0.67	−0.05

All values are median,Q1–Q3; *p* ≤ 0.012 (Benjamini-Hochberg corrected significance level); *Z Z*-value, *PT* physical therapy, *OT* occupational therapy, *Appts* appointments

For individuals with paraplegia, 20 min of time outside of therapy was perceived to be higher intensity activity. Investigation of the subcomponents of time spent outside of PT and OT sessions reveals that ~50% of all time was spent engaged in leisure time sedentary activity. ADLs accounted for a further 37% of time. The amount of time spent in leisure time physical activity and in physically active group classes at a higher self-reported intensity amounted to a median value of 0 min (Table 3).

For individuals with tetraplegia, 22 min of the total waking hours outside of therapy were perceived to be higher intensity activity. We found that ~45% of all time was spent engaged in leisure time sedentary activity and ADLs accounted for ~38% of time. A negligible amount of time was spent in leisure time physical activity and physically active group classes at a higher intensity, with a reported median value of 0 min at admission and discharge (Table 4).

### Instrumented measures of physical activity

**Fig. 4:**
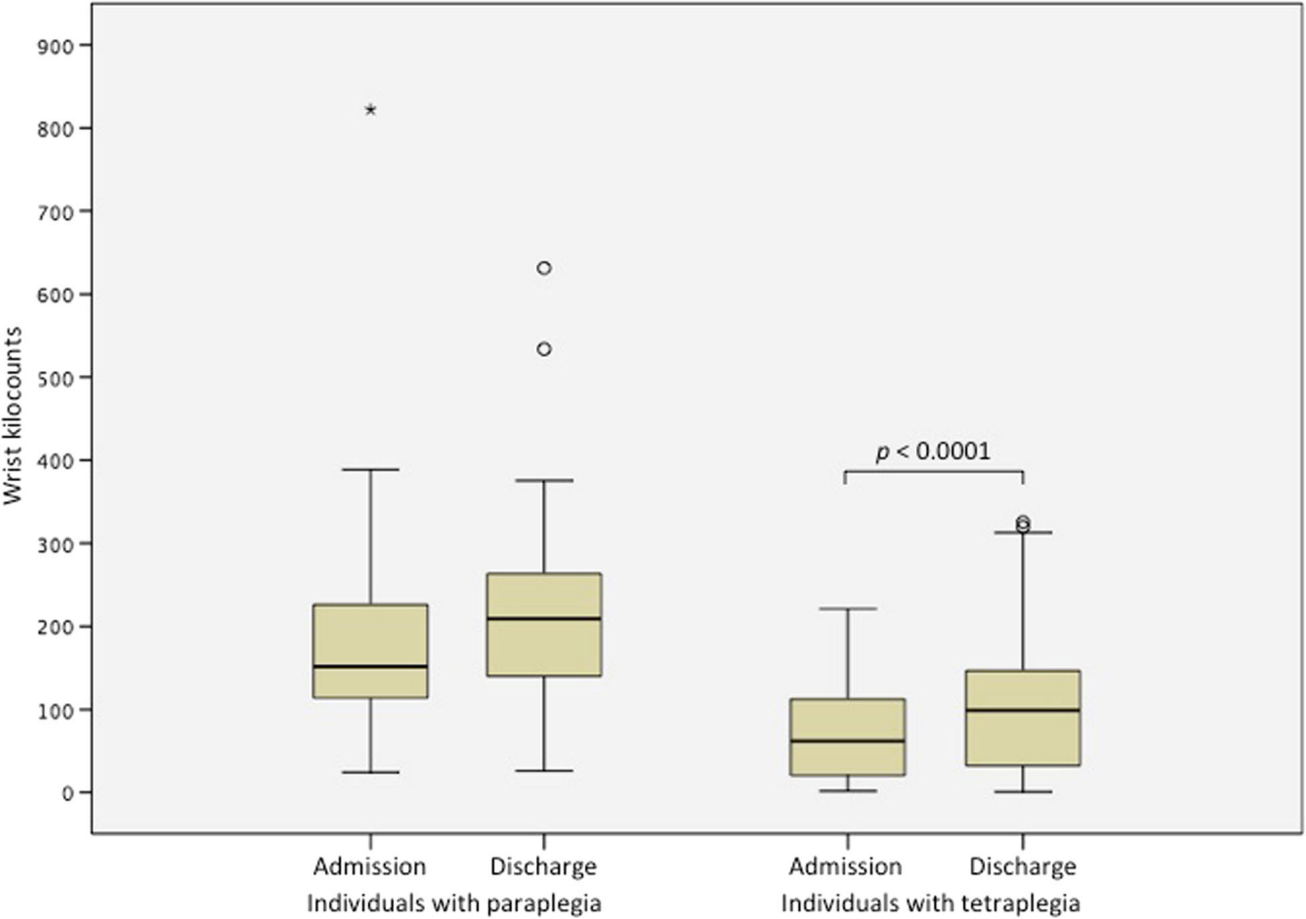
Wrist accelerometry counts at admission and discharge from inpatient rehabilitation

Individuals with paraplegia accrued two or more times the upper extremity physical activity counts measured by wrist accelerometers compared to individuals with tetraplegia (Table 5). Activity kilocounts outside of PT and OT for individuals with paraplegia did not change significantly from admission (151 kilocounts) while individuals with tetraplegia experienced a significant increase in kilocounts during time outside of therapy (Fig. 4).

**Table 5:** Wrist accelerometry and step counts at admission and discharge from inpatient rehabilitation

	Admission	Discharge	*Z value*	*p*	effect size
Particiapants with paraplegia					
Wrist Accelerometry: Total Day	194, 135–258	227, 165–305	~	~	~
Wrist Accelerometry: Outside PT/OT only	151, 112–229	209, 138–273	−2.29	0.022	−0.22
Participants with tetraplegia					
Wrist Accelerometry: Total Day	75, 29–141	112, 35–183	~	~	~
Wrist Accelerometry: Outside PT/OT only	62, 20–113	99, 29–148	−4.02	<0.0001*	−0.44
Ambulatory Participants					
Hip Accelerometry: Total Day	63, 0–726	1488, 236–3593	~	~	~
Hip Accelerometry: Outside PT/OT only	0, 0–662	1097, 176–3130	−3.98	<0.0001*	−0.49

Wrist accelerometry values reported in kilocounts; Hip accelerometry values reported as steps; All values are median, Q1–Q3; *p ≤ 0.012 (Benjamini-Hochberg corrected significance level), *PT* physical therapy; *OT* occupational therapy

**Fig. 5:**
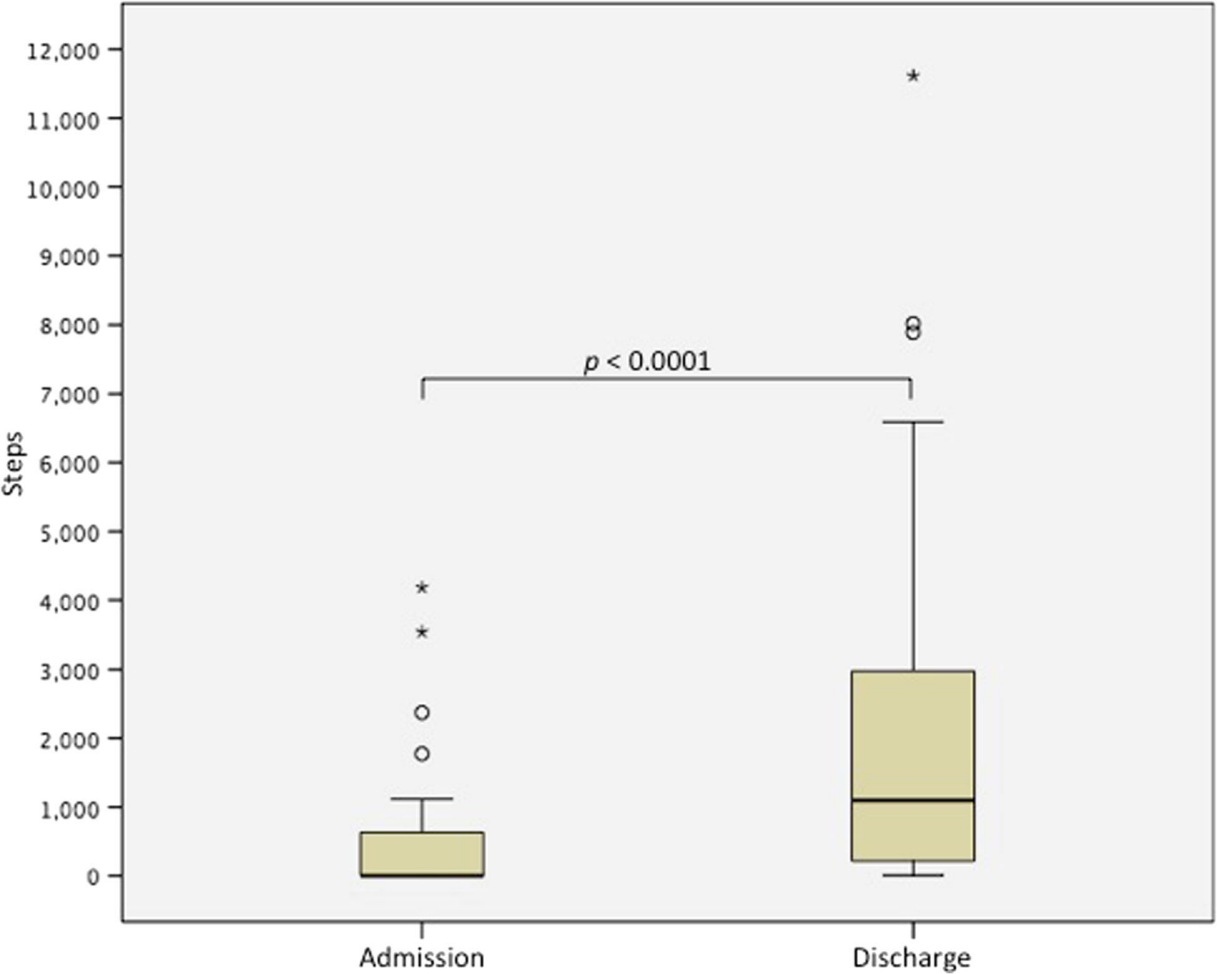
Step counts at admission and discharge from inpatient rehabilitation

For the subset of individuals who were ambulatory (Table 5, Fig. 5), walking steps measured by hip accelerometry increased significantly during time outside of therapy.

## Discussion

### Self-reported physical activity

Time outside therapy constitutes the majority of a participant’s waking hours and herein lies a substantial opportunity for increasing physical activity. Regardless of plegia type or measurement time, ADLs and appointments/educational classes account for ~45% of time that is a necessary and unchangeable part of the day. However, the largest portion of the day is composed of leisure time sedentary activity (watching television, reading, playing games, socializing, etc.), which made up between 235 and 290 min or between 45 and 50% of time. In other words, there are about 4 h in a day potentially available for participants to engage in further physical activity.

While it is recognized that inpatient rehabilitation provides an opportunity to gradually increase physical activity levels in a supervised setting, one goal could be to reach SCI specific physical activity guidelines that recommend individuals accumulate, at a minimum, 20 min of moderate to vigorous intensity aerobic activity twice per week to receive cardiovascular benefit, and an additional two times per week on muscle strengthening [23]. Notably, our data shows that individuals perceive that they are engaged in moderate and heavy intensity activity for a median 20 to 28 min outside of physical and occupational therapy. However, the biggest contributor of this time is the median 7 to 10 min of higher intensity ADLs, and previous research shows that ADLs do not adequately challenge cardiovascular fitness in persons with SCI [4, 34, 35]. This is likely due to the nature of ADLs; they occur most often in very short bouts. Current guidelines indicate that physical activity bouts must be ≥10 min to confer cardiovascular benefit [22, 36].

Just prior to returning to the community, participants were spending a median ~10 min on leisure time physical activity of any intensity with over half of participants reporting no leisure time physical activity whatsoever. Our findings reflect those of Martin Ginis et al. [37] who showed that half of community dwelling individuals with chronic SCI reported no leisure time physical activity. Given the low levels of leisure time physical activity undertaken during inpatient rehabilitation, it is not surprising then, that most individuals do not go on to have active lives. Perhaps an introduction and greater focus on developing leisure time physical activity skills and interests during the inpatient stay would help to foster habits that lead to a physically active lifestyle once participants leave the hospital.

The amount of self-reported physical higher intensity physical activity was similar for individuals with paraplegia and tetraplegia. This observation may be due to fact that individuals perceived most of their day to be sedentary, and thus, the residual physical function did not impact the level of physical activity.

### Instrumented measures of physical activity

Van Hedel et al. [38] found that a gait speed of 0.44 m/s was predictive of the ability of individuals with SCI to walk outdoors with a walking aid, and hence, was considered a threshold for community ambulation. With a median gait speed of 0.77 m/s near discharge (and with only 8 participants less than 0.44 m/s), the majority of our ambulatory participants would have potential for community ambulation. The ambulatory participants demonstrated a median 1097 daily steps by discharge measured by the accelerometers which is at the lower range of daily steps that individuals living with disability and/or chronic illness demonstrate (average of 1200–8800 steps/day) [39]. However, the variability was large in our cohort with 1/3 of individuals with fewer than 500 daily steps. While such variability is attributed in part due to differences in functional ability and variability in accelerometry-measured activity [18], a lack of encouragement or opportunities to accrue walking steps will also influence the levels of physical activity. Measuring walking steps during structured therapy or outside of therapy is not part of usual practice. In the future, non-obtrusive measures such as accelerometers could enable clinicians and patients to monitor and progress physical activity levels, and compare with targets, as well as with existing physical activity guidelines: individuals accumulating less than 5,000 steps/day are classified as sedentary [40, 41]. Such values will also serve as baseline activity from which to build novel clinical trials to increase physical activity within the rehabilitation setting.

Individuals with paraplegia maintain innervation of the upper extremities and, as expected, undertake more upper extremity physical activity as measured by wrist accelerometers than individuals with tetraplegia. The more prevalent use of manual wheelchairs among those with paraplegia relative to those with tetraplegia is likely a large contributor to this difference. Individuals with tetraplegia increased their upper extremity physical activity significantly from admission to discharge commensurate with improvements in clinical measures.

### Self-report versus instrumented measures of physical activity

The accelerometers at the hip and wrist were sensitive to detecting an increase in physical activity over the inpatient stay suggesting that individuals are more active at discharge compared to admission. The finding that accelerometers could detect differences in physical activity over the inpatient stay, as well as between individuals with paraplegia and tetraplegia, provides validity to this technology as an easy and unobtrusive method to measure physical activity [18]. However, individuals do not indicate an increase in physical activity through self-report methods. This discrepancy may be due to recall bias inherent within self-report measures. Alternatively, the fact that individuals are doing more physical activity without any major perception of this suggests that individuals have the capacity to increase their levels of physical activity over the inpatient stay.

### Limitations

Accelerometry may underestimate the number of steps particularly near admission for individuals with SCI who are learning to walk again and steps are very slow [42]. On the other hand, some movements that are recorded via accelerometry can give a ‘false positive’ of the participant doing activity (e.g., assisted transfer).

It has been shown that a ‘wear effect’ may occur when wearing accelerometers such that individuals are more active when the monitors are worn [43]. We believe this potential was minimized in our study due to the regimented nature of participant’s days in an inpatient setting.

Individuals tend to overrate their physical activity participation during recall questionnaires [44]. Furthermore self-report can be unreliable due to poor memory and limited insight. We believe we minimized some issues of recall by administering the PARA-SCI at the end of each day with a trained researcher to facilitate the individual to recollect the events of the day.

## Conclusion

Physical activity is low outside of structured therapy sessions with half of the time spent on sedentary leisure activities. Increases in physical activity over the inpatient stay were detected using hip and wrist accelerometers (representing walking and upper extremity physical activity, respectively), but not using self-report measures, suggesting that individuals may have the capacity to increase their levels of physical activity over the inpatient stay. Our recommendation to increase physical activity outside of PT and OT sessions involves changes in informal and structured exercise. We suggest strategies such as recreational therapy or behavioural counseling to develop leisure time physical activity skills and interests during the inpatient stay which foster habits that lead to a physically active lifestyle once participants leave the hospital. We also suggest the addition of a group class focused on movements that elicit an increase in heart rate to a moderate or heavy intensity for 20 min. Participation in such a class may be enhanced by ensuring that the class is a recognized part of the rehabilitation program (as with PT and OT sessions), by promoting the social aspects of group classes, and providing the various modalities, assistance, and infrastructure necessary to engage individuals with different levels of functional ability.

## Abbreviations

10MWT: 10 Meter walk test
ADL: Activity of daily living
GRASSP: Graded redefined assessment of strength, sensibility and prehension
OT: Occupational therapy
PARA-SCI: Physical activity recall assessment for people with spinal cord injury
PT: Physical therapy
SCI: Spinal cord injury
WISCI II: Walking index for spinal cord injury
